# Bibliometric analysis of the global scientific production on machine learning applied to different cancer types

**DOI:** 10.1007/s11356-023-28576-9

**Published:** 2023-08-11

**Authors:** Miguel Angel Ruiz-Fresneda, Alfonso Gijón, Pablo Morales-Álvarez

**Affiliations:** 1 Department of Microbiology, University of Granada, Granada, Spain; 2 Department of Computer Science and Artificial Intelligence, University of Granada, Granada, Spain; 3 Research Centre for Information and Communication Technologies (CITIC-UGR), University of Granada, Granada, Spain; 4 Department of Statistics and Operations Research, University of Granada, Granada, Spain

**Keywords:** Machine learning, Cancer, Bibliometric analysis, Artificial intelligence, Public health

## Abstract

Cancer disease is one of the main causes of death in the world, with million annual cases in the last decades. The need to find a cure has stimulated the search for efficient treatments and diagnostic procedures. One of the most promising tools that has emerged against cancer in recent years is machine learning (ML), which has raised a huge number of scientific papers published in a relatively short period of time. The present study analyzes global scientific production on ML applied to the most relevant cancer types through various bibliometric indicators. We find that over 30,000 studies have been published so far and observe that cancers with the highest number of published studies using ML (breast, lung, and colon cancer) are those with the highest incidence, being the USA and China the main scientific producers on the subject. Interestingly, the role of China and Japan in stomach cancer is correlated with the number of cases of this cancer type in Asia (78% of the worldwide cases). Knowing the countries and institutions that most study each area can be of great help for improving international collaborations between research groups and countries. Our analysis shows that medical and computer science journals lead the number of publications on the subject and could be useful for researchers in the field. Finally, keyword co-occurrence analysis suggests that ML-cancer research trends are focused not only on the use of ML as an effective diagnostic method, but also for the improvement of radiotherapy- and chemotherapy-based treatments.

## Introduction

The thriving development in sensor and storage technology has enabled the collection of ever-increasing amounts of data (Shilo et al. 2020; Nathan et al. 2022). This growth in data availability is affecting many different fields of research, well beyond computer science and engineering. For example, the increasing digitalization of medical tests such as X-rays, biopsies, electrocardiograms, and blood tests is laying the foundations for personalized medicine (Vokinger and Gasser 2021; Houssein et al. 2021). Likewise, thousands of satellites daily provide unprecedented data streams as remote sensing images for earth-observation and climate change analysis. Also, the evaluations provided by customers in platforms such as Amazon or Netflix generate plenty of relevant information for marketing campaigns.

All this raw data is not enough on its own to make decisions. Indeed, the data must be analyzed by an expert, i.e., a clinician, an environmental scientist, or a business administrator in the three examples above, respectively. Due to the huge amount of available data, this is a daunting task for a single expert, or even for a team of them. The goal of artificial intelligence (AI) is to automate this process of knowledge extraction from data. Early approaches in AI were based on expert systems (Shortliffe 1986; Duan et al. 2005). The idea in expert systems is to hard-code the knowledge of the expert in some formal language so that the machine can apply it. Although this paradigm has proved useful for very structured tasks, it struggles in other problems such as general object and speech recognition, which require subjective and subtle knowledge that cannot be easily codified in formal computer languages (Saibene et al. 2021).

Machine learning (ML) has emerged as a different paradigm to extract knowledge from data (Ravuri et al. 2018; Hameed et al. 2020). Instead of hard-coding the knowledge of an expert, ML algorithms try to learn their own knowledge based on specific examples of the task at hand (Murphy 2012; López-Pérez et al. 2021). This has led to much more accurate results, since ML algorithms learn to extract relevant features and are able to reason based on them (Goodfellow et al. 2016). For instance, consider the problem of cancer detection in histopathological images. An expert-system tries to codify the knowledge of a pathologist into a sequence of instructions that can be systematically applied by a computer (e.g., looking for regions with a certain color or shape). Alternatively, ML algorithms are shown several examples of (labeled) cancerous and non-cancerous images, and the algorithm learns its own rules to make predictions (López-Úbeda et al. 2020).

Many different algorithms have been developed in the machine learning community. One of the most popular approaches is given by artificial neural networks, also known as deep learning, which leverage several layers of simple operations to extract increasingly abstract features that can be used for reasoning (LeCun et al. 2015). For example, convolutional neural networks have achieved astonishing results in image processing, and recurrent neural networks have excelled at speech recognition. Another important family of algorithms is given by probabilistic kernel methods, such as support vector machines (SVM) (Akay 2009; Chen et al. 2011) and Gaussian processes (GP) (Wang et al. 2019; Morales-Álvarez et al. 2022). The latter has become increasingly popular due to its capability to quantify uncertainty, which is essential for real-world applications of machine learning.

Although machine learning has been used in many different applications, in this paper, we focus on the medical domain. More specifically, we are interested in the problem of cancer, which has been studied through machine learning from different perspectives. For example, digital or computational pathology leverages ML algorithms to detect the presence of cancer in digitalized biopsies (Peng et al. 2022; Nguyen et al. 2022; Ain et al. 2022). The goal here is to speed up the cancer detection process, to ensure the democratization of early cancer diagnosis. Machine learning is also used for basic cancer research, in order to analyze the properties of molecules and drugs that can lead to potential treatments (Vamathevan et al. 2019). Likewise, ML algorithms are deployed to improve the treatment of oncology patients, analyzing the results of tumor markers throughout the radiotherapy and chemotherapy processes (Cuocolo et al. 2020). It is important to stress out that cancer is one of the main challenges for the XXI century, as it is the second leading cause of death worldwide according to the American Cancer Society (10 million deaths in 2020 were attributed to cancer) (American Cancer Society 2021).

In order to evaluate and optimize the current huge investment in ML for cancer research, the total volume of scientific production in the field must be analyzed. Bibliometric data analyses can be very useful in the understanding and classification of such a large amount of published documents and can shed light on the development of both ongoing and new research. The aim of the present study is to analyze global scientific production, impact, and research trends on ML applied to the types of cancer that present the highest incidence (in terms of death rate). Many previous bibliometric reviews have focused on specific cancers types, such as breast cancer (Salod and Singh 2020; Joshi et al. 2021), rectal and colorectal tumors (Wang et al. 2020; Kennion et al. 2022), or gynecological ones (Fiste et al. 2022). Whereas these works cover individual cancer types in depth, they do not provide a global unified bibliometric analysis of the most frequent ones. Other works have focused on literature related to specific stages of cancer disease, regardless of the cancer type, such as cancer rehabilitation (Tschuggnall et al. 2021) and cancer survival prediction (Deepa and Gunavathi 2022). There also exist insightful reviews on the most popular ML techniques for cancer research (see Maurya et al. 2023; Mokoatle et al. 2023). But notice that these works focus on the methodological aspects of the ML approaches and do not provide a bibliometric perspective of the field. In contrast to previous work, here we present a unified, novel, updated, and comparative quantitative study for each one of the most important cancers in the last years according to the World Health Organization (lung, colorectal, liver, stomach, and breast cancer). The results presented here are expected to encourage international collaborations between countries and research institutions and to favor the development of new research in the field.

The rest of this paper is organized as follows: The “Methods” section introduces the research methodology, including the search strategies as well as the data processing and analysis. The “Results and discussion” section presents and discusses the main results on the scientific production of machine learning applied to cancer. Finally, the “Conclusions” section summarizes the main conclusions of this work.

## Methods

**Fig. 1:**
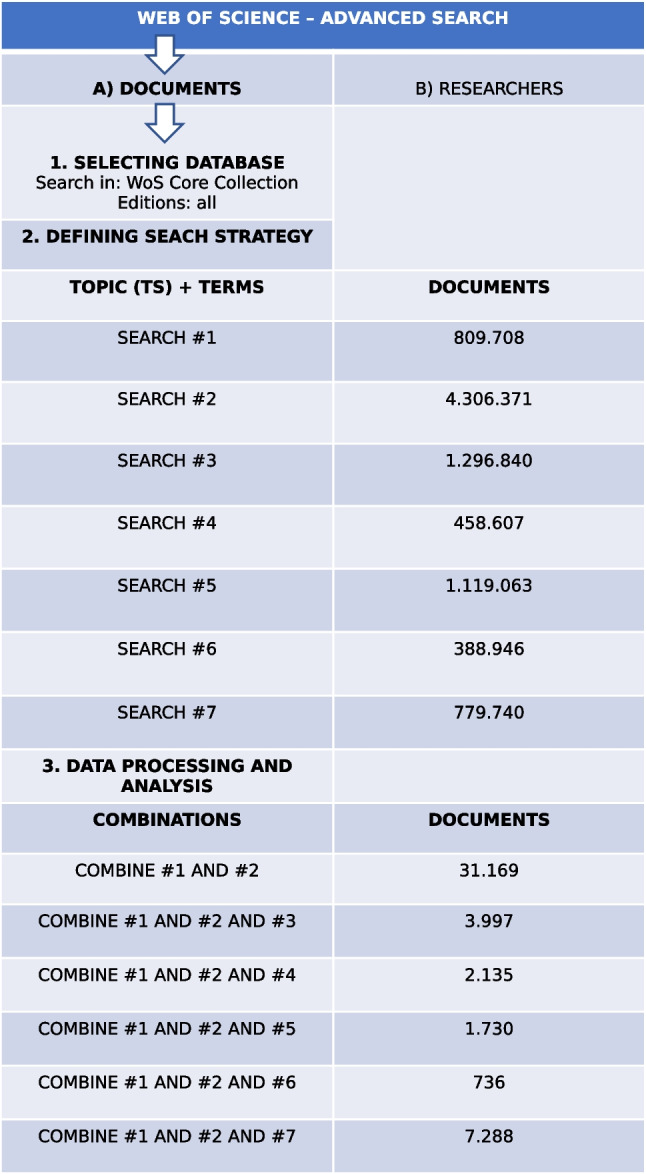
Flow diagram summarizing the search strategy and analysis performed using the Web of Science (WoS)

We have gathered our data from scientific production indexed in the Web of Science core collection databases (WOS 2022). This multi-disciplinary international source references the most prestigious scientific publications in the world and is an essential starting point for bibliometric studies providing indicators of production and scientific impact. We launched our searches from 1900 to 31-12-2021 comprising almost all year’s timespan. The search flow is shown in Fig. 1, where different combinations and number of documents are included.

### Search strategies

To gather data comparing scientific production on machine learning for the study of different types of cancer, we conducted searches in WOS “Web of science core collection”>*Advanced Search*>*TS=Topic*, as summarized in Fig. 1. Topic search strategy includes title, abstract, author keywords, and keywords plus.

Firstly, we designed a general search of machine learning research on cancer over time. For this purpose, the #1 search was performed to identify documents that studied machine learning in general terms by using the equation: *TS*=(“Machine Learning” *OR* “Data Science” *OR* “Machine Intelligence” *OR* “Data mining” *OR* “Big data” *OR* “Artificial Intelligence” *OR* “Deep Learning” *OR* “Deep Learn” *OR* “Supervised Learning” *OR* “Unsupervised Learning” *OR* “Neural networks” *OR* “Convolutional Neural Network” *OR* “Reinforcement Learning” *OR* “Natural Language Processing” *OR* “Natural Language Process” *OR* “Artificial neural network”). *TimeSpan=*1900-2021. After that, the #2 search was performed using the equation: *TS*=(“Cancer*” *OR* “tumor*” *OR* “neoplasia*” *OR* “neoplasm*” *OR* “oncology” *OR* “metastasis” *OR* “metastatic” *OR* “carcinoma”). *TimeSpan*=1900-2021. To identify within the set of documents retrieved in #1, those that studied cancer, we constructed the intersection between the search strategies #1 and #2, by using “Combine #1 *AND* #2”.

Subsequently, we designed a search comparing the use of machine learning for certain types of cancer. Specifically, we focused on types of cancer that caused the highest number of death in 2020, according to the World health Organization (WHO): lung cancer (1.8 million deaths), colon and rectum cancer (916,000 deaths), liver cancer (830,000 deaths), stomach cancer (769,000 deaths), and breast cancer (685,000 deaths). As a results, the #3 search included a list of terms about lung cancer: *TS*=(“lung” *OR* “pulmonary” *OR* “pulmonic”). *TimeSpan*=1900-2021. The search #4 was constructed for colon and rectum cancer: *TS*=(“colon” *OR* “rectum” *OR* “colorectal” *OR* “large intestine”). *TimeSpan*=1900-2021. The #5 search included liver cancer terms: *TS*=(“liver” *OR* “hepatocellular” *OR* “hepatoma”). *TimeSpan*=1900-2021. Finally, the #6 search was constructed for stomach cancer: *TS*=(“stomach” *OR* “gastric”) (*TimeSpan*=1900-2021), and the #7 search for breast cancer: *TS*=(“breast”). *TimeSpan*=1900-2021.

### Data processing and analysis

Data obtained from the search “Combine #1 *AND* #2” were tabulated, and we produced a table of annual scientific production on machine learning applied to cancer studies by institution, country, and journal. The 31,169 reported documents resulting from the search “combine #1 *AND* #2” were processed and standardized in Excel.

For the individual analysis of each cancer type resulting from the searches “combine #1 *AND* #2 *AND* #3,” “combine #1 *AND* #2 *AND* #4,” “combine #1 *AND* #2 *AND* #5,” “combine #1 *AND* #2 *AND* #6,” “combine #1 *AND* #2 *AND* #7,” we designed a database to analyze the production and impact of the studies recorded about machine learning, considering TSP (total studied produced), CR (citations received), MCS (mean citations/study), CS (citing studies), +CS (citations received by the most cited work), and H-index (number of studies that have received the same or a higher number of citations). Additionally, we designed a database to analyze the top 5 production of the studies recorded disseminated by institutions, producer countries, and journals. Finally, visualization network mapping for co-occurrence keywords was performed for each cancer type to analyze the global trends on the topic. To visualize the bibliometric networks, we used VOS-viewer software (https://www.vosviewer.com/), which works with units of analysis (authors, organizations, keywords, etc.) and of measurement (links, frequency, centrality, distance), to illustrate our results by grouping similarities in clusters. To build the co-occurrence networks, we generated vectors, which were pre-displayed in PAJEK (http://mrvar.fdv.uni-lj.si/pajek/), with definitive drawings created in VOS-viewer. We used this process because VOS-viewer is limited in that it labels nodes based on an internal, non-modifiable schedule. We labeled as many nodes as possible while guaranteeing the set were correctly displayed.

## Results and discussion

In this section, we present and discuss our main results. We first study the more general field of machine learning applied to any cancer type (see “Overview of scientific production on machine learning applied to cancer”). Then, we separately focus on the five cancer types with highest incidence (see “Comparison of scientific production on different types of cancers” and “Analysis of keywords co-occurrence on different types of cancers”). Finally, based on the literature identified in this section, we briefly discuss the potential of machine learning in cancer research (see “Machine learning in cancer research: a paradigm shift in diagnosis, treatment, and beyond”).

### Overview of scientific production on machine learning applied to cancer

**Fig. 2:**
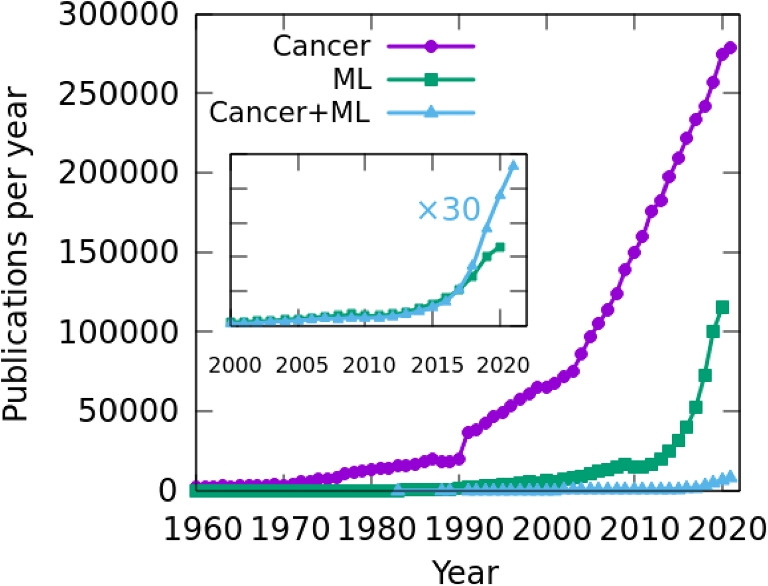
Number of publications per year on cancer (violet), ML (green), and ML applied to cancer (blue), over time. The inset compares the ML and Cancer+ML cases, where the latter has been linearly scaled by a factor of 30 for a better visualization

Our search showed a high amount of studies published on machine learning applied to cancer research until 2021, with a total of 31,169 documents (see Table 1). The first study applying ML to cancer was published in 1983 as a meeting abstract in the journal Medical Physics, with the title “An artificial-intelligence program to plan radiotherapy for cancer of the oral cavity” (Paluszynski et al. 1983). However, a solid interest was not observed until 3 decades later, when an exponential increase in the number of publications occurred in the decade of 2010. In fact, 75% of the documents (23,517 out of 31,169) have been published in the last 5 years analyzed (2017–2021). This may be due to the rapid development and advancement of ML in recent years in both computational resources and algorithms (Jordan and Mitchell 2015), as suggested in Fig. 2, where an increasing number of publications appear within that period. Besides, in the inset of the same figure, we clearly observe a correlation between the trends in the scientific production on ML only and ML applied to cancer, which indicates that ML techniques have been applied to cancer since their early days. Until 2017, the production in ML (green line) was approximately equal to 30 times the production in Cancer+ML (blue line), while from 2018 onward, the latter topic has grown even more rapidly in relative terms than general ML.

**Table 1:** TSP (total studies produced) per year on ML applied to cancer since 1983 until 2021

Year	TSP	Year	TSP	Year	TSP
1983	1	2000	120	2021	460
1988	4	2001	118	2013	557
1989	1	2002	149	2014	709
1991	12	2003	211	2015	888
1992	23	2004	227	2016	1180
1993	16	2005	276	2017	1770
1994	36	2006	320	2018	2895
1995	40	2007	376	2019	4750
1996	52	2008	355	2020	6342
1997	97	2009	414	2021	7760
1998	112	2010	388		
1999	89	2011	421	Total	31,169

**Table 2:** TSP (total studies produced) and TSP percentage of the total of 31,169 studies produced, classified by institution, country, and journal

Institution	TSP	%TSP	Country	TSP	%TSP	Journal	TSP	%TSP
Harvard University	926	2.97	USA	10,051	32.25	Proceedings of Spie	826	2.65
University of California System	885	2.84	China	6271	21.56	Lecture Notes in Computer Science	668	2.14
University of Texas System	860	2.76	India	2275	7.30	Medical Physics	572	1.84
Chinese Academy of Sciences	619	1.99	England	2015	6.47	Scientific Reports	552	1.77
Harvard Medical School	501	1.61	Germany	1744	5.60	IEEE Access	445	1.43

In turn, the need to find new methods to improve the diagnosis and treatment of a disease as serious as cancer (Jemal et al. 2011) has triggered a growing number of publications since the last century (see Fig. 2). The relevance of cancer has also motivated the application of ML techniques in that field as a useful tool. The importance of ML research applied to cancer can be appreciated by comparing the total scientific production that we have detected in the present study compared to the use of ML in other fields. Bibliometric studies analyzing applications of ML in physiological signals (Faust et al. 2018), sustainable manufacturing (Jamwal et al. 2021), Industry 4.0 (Muhuri et al. 2019), public health problems (dos Santos et al. 2019), maritime industry (Munim et al. 2020), management of depressive disorders (Tran et al. 2019), cybernetics (Xu et al. 2019), or operations environment (Dhamija and Bag 2020) found a total of 53, 96, 194, 250, 279, 544, and 1854 publications, respectively, far from the huge amount of 31,169 documents analyzed in the present study. Notice that the lower number of articles found by these authors could be also influenced by the use of a different search strategy, including different selection filters, search periods, types of documents, and database collection, for example.

**Table 3:** Bibliometric parameters classified by cancer type

Cancer type	TSP	CR	MCS	CS	+CR	H-index
Lung	3997	69,640	17.42	48,651	3826	110
Colon and rectum	2135	30,392	14.24	22,893	1181	76
Liver	1730	23,416	13.54	19,308	679	62
Stomach	736	8662	11.77	6515	261	43
Breast	7288	128,171	19.59	84,637	3826	137

*TSP* total studies produced, *CR* citations received, *MCS* mean citations/study, *CS* citing studies, *+CR* citations received by the most cited work, *H-index* number of studies that have received the same or a higher number of citations

Regarding the nationality of the institutional affiliation, US institutions are the leaders of the field with 4 institutions in the top 5: Harvard University (926 studies), University of California System (885 studies), University of Texas System (860 studies), and Harvard Medical School (501 studies), as can be seen in Table 2. Only one from China (The Chinese Academy of Science) is among the top five (fourth position) with 619 studies. This fact indicates the clear dominance and potential of American institutions on the investigated subject. However, it can be observed that the production is very distributed, since the institution with the most publications on the subject (Harvard University) accounts for 2.97% of the total number of published works (see Table 2), and the rest of the institutions present values that are not very different. According to these results, it is logical that in the ranking by countries, the leader is the USA, with a total of 10,051, which represents a huge amount of 32.25% of the total production. China follows with a total of 6721. India also stands out in this sector with 2375 documents (7.30%), which can be related to the great development gained in research in the field of medicine and pharmacology in recent years (Meena and Mathaiyan 2021). In terms of journals, the production is very distributed since none in particular includes the majority of studies. The one that published the most papers on the subject is Proceedings of Spie, with 826 and a total of 2.65% of the production. It should be noted that the 3 most producing journals are in the field of computer science, while the rest of the top 5 are journals dedicated to medicine.

### Comparison of scientific production on different types of cancers

Following the analysis of the global scientific production on machine learning applied to cancer addressed in the last section, a specific and individualized study was carried out on different types of cancer with high incidence and mortality rates (lung, colon, liver, stomach, and breast cancer). The aim of such an analysis was to elucidate for the first time the differences or similarities, in terms of scientific production, between different types of cancer in order to obtain potentially relevant conclusions. Specifically, we analyze the scientific impact and production at the level of institutions, countries, and scientific journals.

Our results analyzing five types of cancer, shown in Table 3, indicated that breast cancer has been the most studied with ML techniques, with 7288 studies, followed by lung, colon, liver, and stomach cancer with 3997, 2135, 1730, and 736, respectively. Studies on breast cancer also have the highest impact with 128,171 total citations and an H-index of 137. Out of the 84,637 citing studies, the one that has received the most citations contains a total of 3826 total citations. Lung cancer is in second position in terms of scientific impact with a total of 69,640 citations, which supposes almost half of those received for breast cancer. As can be observed, colon and liver cancer present intermediate levels of impact in third (30,392) and fourth position (23,416), respectively. In last place is stomach cancer with 8662 citations received, well below the rest of cancer types.

**Table 4:** Scientific production on ML applied to different types of cancer classified by institution, country, and journal

Cancer Type	Institution	TSP	%TSP	Country	TSP	%TSP	Journal	TSP	%TSP
Lung	Harvard Univ	142	3.55	USA	1327	33.20	Medical Physics	127	3.18
	Univ. of California System	119	2.98	China	1117	27.95	Proceedings of Spie	93	2.33
	Univ. of Texas System	111	2.78	India	311	7.78	Scientific Reports	88	2.20
	Chinese Academy of Sciences	91	2.28	England	206	5.15	Frontiers in Oncology	85	2.18
	Harvard Medical School	83	2.08	South Korea	199	4.98	IEEE Access	73	1.83
Colon and rectum	Harvard Univ	95	4.45	USA	667	31.24	Proceedings of Spie	47	2.20
	Harvard Medical School	64	3.00	China	482	22.58	Scientific reports	45	2.11
	Univ. of California System	60	2.81	England	185	8.67	Cancers	36	1.69
	Univ. of Texas System	53	2.48	Japan	161	7.51	Lecture Notes in computer Science	36	1.69
	Massachusetts General Hospital	48	2.25	Germany	137	6.41	Medical Physics	32	1.50
Liver	Sun Yat Sen Univ	62	3.58	China	635	36.71	Frontiers in Oncology	45	2.60
	Chinese Academy of Sciences	58	3.35	USA	454	26.24	Medical Physics	34	1.97
	Fudan Univ	53	3.06	Japan	116	6.71	Scientific Reports	33	1.91
	Univ. of Texas System	51	2.95	India	106	6.13	Proceedings of Spie	29	1.68
	Zhejiang Univ	47	2.72	Germany	96	5.55	Cancers	27	1.56
Stomach	Chinese Academy of Sciences	28	3.80	China	297	40.35	Scientific Reports	20	2.72
	National Cancer Center Japan	21	2.86	USA	127	17.26	Gastrointestinal Endoscopy	18	2.45
	Shangai Jiao Tong Univ	20	2.72	Japan	110	14.95	World Journal of Gastroenterology	17	2.31
	Univ. Chin. Academy of Sci	20	2.72	South Korea	74	10.05	Digestive Endoscopy	14	1.90
	Univ. of Tokyo	20	2.72	Germany	36	4.89	Frontiers in Oncology	12	1.63
Breast	Harvard Univ	177	2.43	USA	2227	30.56	Proceedings of Spie	297	4.08
	Univ. of Texas System	170	2.33	China	1271	17.44	Lecture Notes in computer Science	197	2.70
	Univ. of California System	163	2.24	India	746	10.24	Scientific Reports	116	1.59
	Chinese Academy of Sciences	124	1.70	England	463	6.35	IEEE Access	103	1.41
	Egyptian Knowledge Bank EKB	117	1.61	Germany	307	4.21	Medical Physics	98	1.35

The total production of studies (TSP) for each type of cancer (see Table 3) matches well with the data on articles with the highest number of citations. Indeed, the works by Ehteshami Bejnordi et al. (2017); Coudray et al. (2018); Ye et al. (2003); Sirinukunwattana et al. (2016); Kather et al. (2019) stand out in the fields of breast, lung, liver, colon/rectum, and gastric cancer with 1291, 1069, 684, 661, and 437 citations, respectively. Based on these highly cited studies and some recent review publications, one can briefly analyze the most popular approaches in each of the explored areas. In the context of lung cancer, popular machine learning methods include convolutional neural networks (CNNs), support vector machines (SVMs), and random forests, which have been employed for tasks such as tumor classification, early detection, and survival prediction using medical images as inputs (Coudray et al. 2018; Li et al. 2022; Wang 2022). The same widely used supervised classifiers are leveraged for breast cancer, sometimes combined with long-short term memory (LSTM) networks (Ehteshami Bejnordi et al. 2017; Zhang et al. 2020). For colorectal cancer, on the one hand, we have studies using CNNs from images (Yu and Helwig 2021; Sirinukunwattana et al. 2016), and on the other hand, we have classification algorithms like random forest to analyze genetic and molecular data, aiding in predicting disease progression and survival outcome (Koppad et al. 2022). Similarly to colorectal cancer, in liver cancer research, there are applications of CNNs to medical images (Othman et al. 2022), but also studies focusing on genetic data which employ random forest (Ye et al. 2003; Hasan et al. 2023). Lastly, in stomach cancer, popular convolutional and recurrent neural networks have been employed for automatic tumor detector from medical images. Kather et al. (2019); Zhao et al. (2022); Niu et al. (2020). These examples highlight the diversity of machine learning techniques that have been applied to different types of cancers, aiding in enhanced diagnosis, prognosis, prediction, and treatment planning.

It is interesting to realize that the 3 types of cancer with the highest impact and scientific production here analyzed in terms of ML research (breast, lung, and colon, in that order) are those with the highest number of cases worldwide in 2019: breast (19.8 million cases), colon and rectum (11.46 million cases), and lung (3.21 million cases) (Roser and Ritchie 2015). This fact indicates that ML-cancer research is applied proportionally to the most prevalent cancers at the present time. Interestingly, this correlation is not so clear between the ML scientific production and the number of deaths per year caused by each cancer type. Indeed, the most investigated cancer with ML tools (breast cancer) only produced a total of 700,660 deaths in 2019 (3.5% annual mortality), whereas colon and lung cancers showed higher mortality rates with 1.09 and 2.04 million deaths, respectively. Another important point with certain influence in the application of ML to cancer research is that ML methods usually serve as early detection and diagnosis techniques through image processing, and this task may perform more or less effectively for some specific cancer types than for others. For example, ML techniques are specially useful to predict lung or breast cancer detection with image processing (LG and AT 2013; Priya and Ramamurthy 2018). In any way, our results clearly show that the higher the incidence of a cancer type is, the greater the effort carried out in terms of ML studies on this type of cancer.

Regarding institutional affiliation (see Table 4), Harvard University leads in the number of publications for lung, colon, and breast cancer with 142, 95, and 177, respectively, being by far the one that published the most papers on the subject. The University of California System, Chinese Academy of Sciences, and the University of Texas System are of great importance as well since they are in the top 5 for up to 4 types of cancer. It is noteworthy that stomach cancer is mainly investigated by Asian institutions from China and Japan, monopolizing the top 5 (Chinese Academy of Sciences, National Cancer Center Japan, Shanghai Jiao Tong University, University Chinese Academy of Sciences CAS, and University of Tokyo). It is interesting to realize that, out of the 2.71 million total cases of stomach cancer in the world in 2019, Asia had an enormous amount of 2.09 million (almost 78% of the total cases) during that year (Roser and Ritchie 2015). In comparison, Europe, Africa, and America present 337,292; 57,830; and 225,947 cases, respectively. This high incidence explains the demonstrated leading role of Japanese and Chinese institutions in this type of cancer. In general terms, American, Chinese, and Japanese institutions account for the majority of research. Only Egypt’s EKB breaks this rule by being the fifth institution with the most number of documents in breast cancer. This fact is surprising, since Egypt is not one of the countries with the highest number of cases.

As expected from the above results, the USA and China have the highest number of published papers, accounting for the majority of the publications. 33.20, 31.24, 26.24, 17.26, and 30.56% of the papers published for lung, colon, liver, stomach, and breast cancer, respectively, belongs to the USA, while China contributes with 27.95, 22.58, 36.71, 40.35, and 17.44%, respectively, of the total number of published studies. The fact that the USA and China are two economic superpowers and invest the most in research and development can explain these results. But, in addition, they are the countries with the highest number of people with cancer in the world from 2017: 22.86 million cases (USA) and 22.42 million cases (China) (Roser and Ritchie 2015). England, India, Germany, and Japan also stand out, although their production is in general lower than the USA and China.

Journals in the field of computer science and medicine are the most commonly used to publish the studies analyzed here. Medical journals such as Medical Physics, Scientific Reports, Frontiers in Oncology, and Gastrointestinal Endoscopy are the most used journals for lung, colon, liver, and stomach cancer. Specifically, the journal Scientific Reports is the only one that appears among the top 5 in all the cancer types analyzed here. However, Proceedings of Spie leads studies for breast cancer. Although medical journals dominate for most of the cancers studied here, in total output, computer science journals have a leading role, thanks to the contribution of the big amount of documents published on breast cancer (Tables 2–3).

Knowing which countries and institutions publish the most in terms of ML applied to cancer is crucial for improving international collaboration between research groups specialized in the field. This study could substantially facilitate the search for expert researchers, groups, and institutions and thus the improvement, development, and advancement of research on this topic. In addition, knowing the journals where these papers are most published can facilitate the process of documentation, submission, and publication in the field.

### Analysis of keywords co-occurrence on different types of cancers

Constructing network maps for co-occurrence keywords allowed us to visualize and evaluate the different global trends for machine learning studies on cancer. Five different network maps, one for each cancer type, were constructed and analyzed. For all the network maps, the minimum number of occurrences of a keyword was set at 30, in order to avoid spurious correlations. Also, the intensity in the correlation between the different keywords was expressed as TLS (total link strength) by Vos-viewer.

**Fig. 3:**
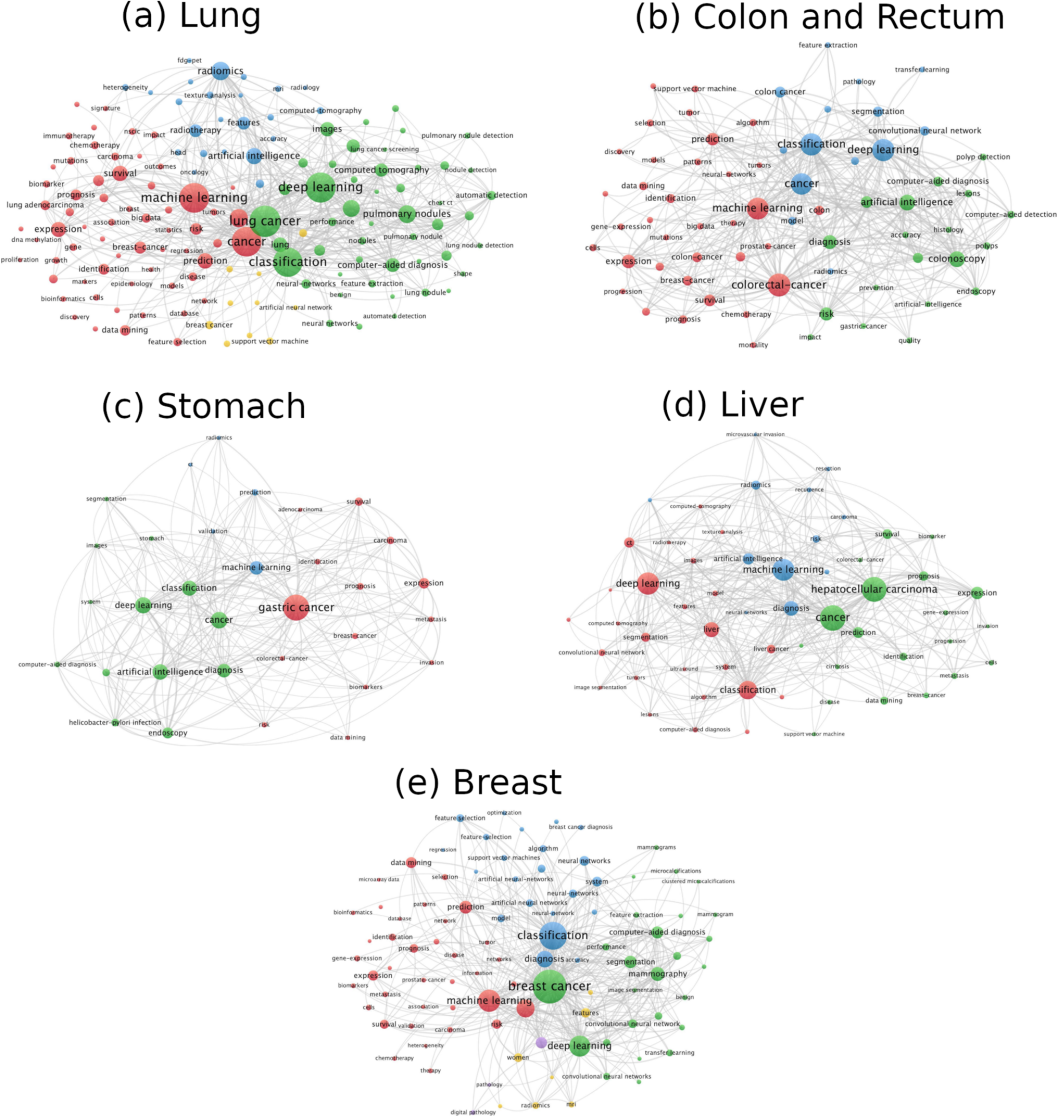
Visualization network map of keywords co-occurrence for all the documents reported for machine learning on **a** lung, **b** colon and rectum, **c** stomach, **d** liver, and **e** breast cancer. The size of the spheres is proportional to the number of occurrences of each keyword. The lines represent the total link strength and the correlation between the keywords

As expected, the most frequent co-occurrence keywords in all cancer types (see Fig. 3) were found to be important technical words in the fields, such as “cancer,” “machine learning,” “deep learning,” and “survival.” However, other frequent co-occurences reveal current trends in ML research applied to cancer. For example, it is worth mentioning “diagnosis” and other related terms such as “computer aided diagnosis,” “detection,” “computer aided detection,” “prognosis,” or “prediction.” These results suggest that ML is mainly used as a diagnosis and prevention method and thus can be employed as an important tool for early detection of tumors, which plays a crucial step for disease treatment (Cruz and Wishart 2006). In addition, the high co-occurrence levels of the keyword “classification” indicated how the application of ML is also important in classifying the cancer disease by type, giving key information about how to proceed for the treatment. “Radiotherapy” and “chemotherapy” present as well a high co-occurrence in all cancer types analyzed here, suggesting that ML techniques are not only useful as an early diagnosis method, but also could play an important role in cancer treatment. Indeed, machine learning is being investigated to enhance the efficiency of radiotherapy treatments against tumors (Meyer et al. 2018; Deist et al. 2018). “Radiomics” is also highlighted as a keyword with high co-occurrence in all the analyzed cancer types. Radiomics is an artificial intelligence-based methodology which uses data-characterization algorithms to extract critical information from medical images through spatial distribution of signal intensities and pixel interrelationships. Many studies have revealed the potential of radiomics to improve clinical decision and radiotherapy workflow (Giraud et al. 2019; van Timmeren et al. 2020). However, still further development is necessary for the implementation of this technique in hospitals.

In the case of stomach or gastric cancer, we highlight the co-occurrence of the keyword *Helycobacter pylori* with a total amount of 43 (TLS=187) (see Fig. 3c). This highlights this bacterium as a major concern in stomach cancer. Indeed, a great number of studies about machine learning on stomach cancer are focused on people infected with the bacterium *H. pylori*, which could play a significant role in cancer development Polk et al. (2010). A similar case was found for liver cancer with the keyword *cirrhosis* (42 co-occurrences and TLS of 163) (Fig. 3d).

The keyword “benign” appeared most frequently for breast cancer with 118 total co-occurrences (TLS=749) in Fig. 3e. This suggests a higher appearance of benign tumors for this cancer type. This fact matches very well with the death rate for breast cancer, which has lower values (8.62 deaths per 100,000 individuals) in comparison with lung (25.18), colon (13.69), and stomach (11.88) cancer (Roser and Ritchie 2015). Interestingly, breast cancer is the only one that differentiates between patient’s gender. The keyword woman reported a total of 233 co-occurrences with a TLS of 1142. However, male gender does not appear as an important keyword on the cancer types analyzed within this work. This suggests most of ML studies on breast cancer are focused on female patients because most cases occur in women.

The co-occurence keyword analysis presented here clearly shows the current trends on machine learning studies in cancer such as the use of this technique in diagnosis, prognosis, detection, prediction, improvement of radiotherapy treatment, radiomics as a promising tool, etc. This analysis strongly supports the potential of these techniques and could be useful to boost further research in the field.

### Machine learning in cancer research: a paradigm shift in diagnosis, treatment, and beyond

Along this section, we have analyzed a vast amount of literature discussing the potential of ML in cancer research. Here, we provide a brief summary of the main challenges and opportunities identified in this field, including ethical considerations. For further details, please refer to the provided references.

**Early detection and accurate diagnosis**. One of the most promising applications is improving early detection and accurate diagnosis. By training models on large-scale datasets comprising imaging data, clinical records, and genetic profiles, ML algorithms can aid in the identification of subtle cancer-related patterns that may escape human detection (McKinney et al. 2020).

**Precision medicine and personalized treatment**. Cancer is a highly heterogeneous disease, requiring tailored treatment approaches for each patient. ML enables the development of predictive models that consider individual patient characteristics, such as genetic variations and lifestyle factors, to guide personalized treatment decisions. By leveraging these models, clinicians can optimize therapy selection, dosage, and scheduling, leading to improved outcomes and reduced adverse effects (MacEachern and Forkert 2021).

**Drug discovery and repurposing**. Traditional drug discovery processes are time-consuming and costly. ML algorithms offer an innovative approach to accelerate the identification of potential therapeutic targets and drug candidates. By integrating large-scale molecular and clinical data, ML models can predict drug efficacy and toxicity, thereby enabling the identification of promising candidates for further experimental validation (Dara et al. 2022).

**Challenges and ethical considerations**. While ML holds immense promise, there are critical challenges that need to be addressed. Issues related to data quality, interpretability of complex models, bias in training datasets, and ethical considerations regarding patient privacy and consent require careful attention. Ensuring transparency, accountability, and equitable access to the benefits of ML-driven cancer research must be central to its implementation (Sorell et al. 2022; Yu et al. 2016).

In conclusion, ML has the potential to revolutionize cancer research. It represents a paradigm shift in our ability to harness the power of data and computational algorithms to confront the challenges posed by cancer. By addressing the associated challenges and ethical considerations, the integration of ML in cancer research holds immense promise for improving patient outcomes and transforming the landscape of cancer prevention and treatment.

## Conclusions

In this work, we investigate the global scientific production and impact of machine learning applied to different cancer types. The huge amount of documents included in our study (more than 30,000), most of them published in the last few years, reveals the great interest raised recently in this field. The high levels of production in the publications that involve ML applications to cancer are a consequence of the combination of a novel and revolutionary technology such as ML and a decades-old research field such as cancer, with a deep impact on society and global health, causing millions of deaths annually.

By means of bibliometric methods, we have carried out a quantitative analysis of the scientific production on ML applied to the five most relevant cancer types (namely, breast, lung, colon/rectum, liver, and stomach cancer). We have confirmed that there exists a correlation between the incidence of different cancer types and the amount of publications that involve machine learning for that type of cancer. When classifying publications by countries, it is clear that the USA and China are the main scientific producers in the general case, but some local correlation can be found between the number of cases and number of publications, as in the case of China and Japan in the study of stomach cancer. Finally, the co-occurrence diagrams show intriguing correlations and point out the present and future trends of ML-cancer research, not only in the use of ML as an effective diagnostic method, but also as a useful tool for improving radiotherapy and chemotherapy-based treatments.

In addition to the interesting relations found when comparing the number of publications and the number of cancer cases, recognizing the countries and institutions that most study the field of ML applied to cancer can be helpful to establish international collaborations. Furthermore, knowledge of the journals where the studies are most published can facilitate access to the appropriate information, as well as the process of submission and publishing in the field. Therefore, this work can serve as a guide for numerous researchers to get insights into the scientific production of ML applied to different cancer types, a remarkably active field due to its implications on global health.

## Data Availability

Not applicable
